# Perforated Jejunal Gastrointestinal Stromal Tumor in a Young Male Patient With Acute Peritonitis: A Case Report and Literature Review

**DOI:** 10.7759/cureus.92325

**Published:** 2025-09-14

**Authors:** Srinivasa Swamy Bandaru, Chaitanya P Garg, Pawan Kumar Sah, Ammar Shahid Tanweer, Majd H Shaheen, Bashayer Alshamsi

**Affiliations:** 1 Surgery, Saqr Hospital, Emirates Health Services, Ras Al Khaimah, ARE; 2 Surgery, Ras Al Khaimah (RAK) Medical and Health Sciences University, Ras Al Khaimah, ARE; 3 Internal Medicine, Ras Al Khaimah (RAK) Medical and Health Sciences University, Ras Al Khaimah, ARE

**Keywords:** acute peritonitis, gastrointestinal stromal tumor, gist, imatinib therapy, jejunal, perforation, surgical resection

## Abstract

Gastrointestinal stromal tumors (GISTs) are rare mesenchymal neoplasms of the gastrointestinal (GI) tract, typically diagnosed in older adults. Jejunal GISTs with spontaneous perforation leading to acute peritonitis are uncommon, particularly in young individuals. We report the case of a 32-year-old male who presented with acute abdominal pain, vomiting, and peritoneal signs. Contrast-enhanced computed tomography (CT) revealed pneumoperitoneum and a heterogeneously enhancing mass at the jejunoileal junction, raising suspicion for a perforated GIST. Emergency exploratory laparotomy confirmed a 6 × 6 cm perforated tumor on the antimesenteric border of the jejunum, which was resected with primary anastomosis. Histopathology confirmed a high-grade spindle-cell GIST with increased mitotic activity. The patient recovered well postoperatively and started on adjuvant imatinib therapy following oncological consultation. A four-week post-discharge positron emission tomography-CT (PET-CT) scan showed no residual disease or metastasis. Perforated jejunal GISTs are rare and carry a high risk of recurrence and poor prognosis if not promptly managed. Surgical resection remains the cornerstone of treatment, with adjuvant tyrosine kinase inhibitor therapy recommended for high-risk cases. Given the aggressive nature of perforated GISTs, long-term surveillance is essential. This case highlights the importance of early diagnosis and prompt surgical intervention in perforated jejunal GISTs, particularly in younger patients. Multidisciplinary management with adjuvant therapy and rigorous follow-up is crucial to optimizing outcomes.

## Introduction

Gastrointestinal stromal tumors (GISTs) represent the most prevalent type of mesenchymal tumors within the gastrointestinal (GI) tract [1,2]. However, they account for only 0.1% to 3% of all GI neoplasms and arise from interstitial cells of Cajal, which are located in the muscularis propria layer of the intestine and are specialized GI cells that act as pacemakers, regulating the rhythmic contractions that move food through the digestive tract [3,4]. The stomach and small intestine are the most affected sites [1,3,4]. GISTs are typically diagnosed at a median age of 65, with occurrence in individuals under 40 being rare, comprising less than 10% of cases [3]. Although all GISTs have malignant potential, the risk of malignancy is primarily correlated with tumor size. In the early stages, these tumors are often asymptomatic unless they reach a considerable size, at which point they may present with clinical manifestations. GI bleeding is the most common complication, followed by obstruction, while perforation remains an uncommon but severe manifestation, as well as the risk of disease dissemination and recurrence [5]. This case report describes a perforated jejunal GIST presenting with acute peritonitis, underscoring the significance of prompt diagnostic evaluation, early surgical intervention, oncological consultation, and adjuvant therapy. Given that perforated high-grade GISTs are classified as advanced disease with an increased risk of recurrence, timely management is crucial.

## Case presentation

A 32-year-old male presented to the emergency department with a five-hour history of acute abdominal pain. The pain began as cramping in the epigastrium before becoming generalized across the entire abdomen. This was associated with four episodes of vomiting. His systems review was otherwise unremarkable. His medical history was significant only for a minor knee surgery several years prior, with no reported complications and no history of chronic disease.

On admission, the patient was conscious and alert with a Glasgow Coma Scale (GCS) of 15/15. Vital signs revealed an elevated blood pressure of 142/90 mmHg, with a normal temperature, pulse rate, respiratory rate, and oxygen saturation. His body mass index (BMI) was calculated to be 39. Abdominal examination demonstrated moderate generalized tenderness without guarding, rigidity, or rebound. Bowel sounds were present, and examination of the hernial orifices and external genitalia was normal.

Initial laboratory investigations showed a borderline elevated white blood cell (WBC) count of 11.0 × 10^9^/L, a hemoglobin level of 14 g/dL, and a significantly elevated C-reactive protein (CRP) level of 182 mg/L. Platelet count, liver function tests, renal function tests, amylase, and lipase were all within normal limits.

**Figure 1:**
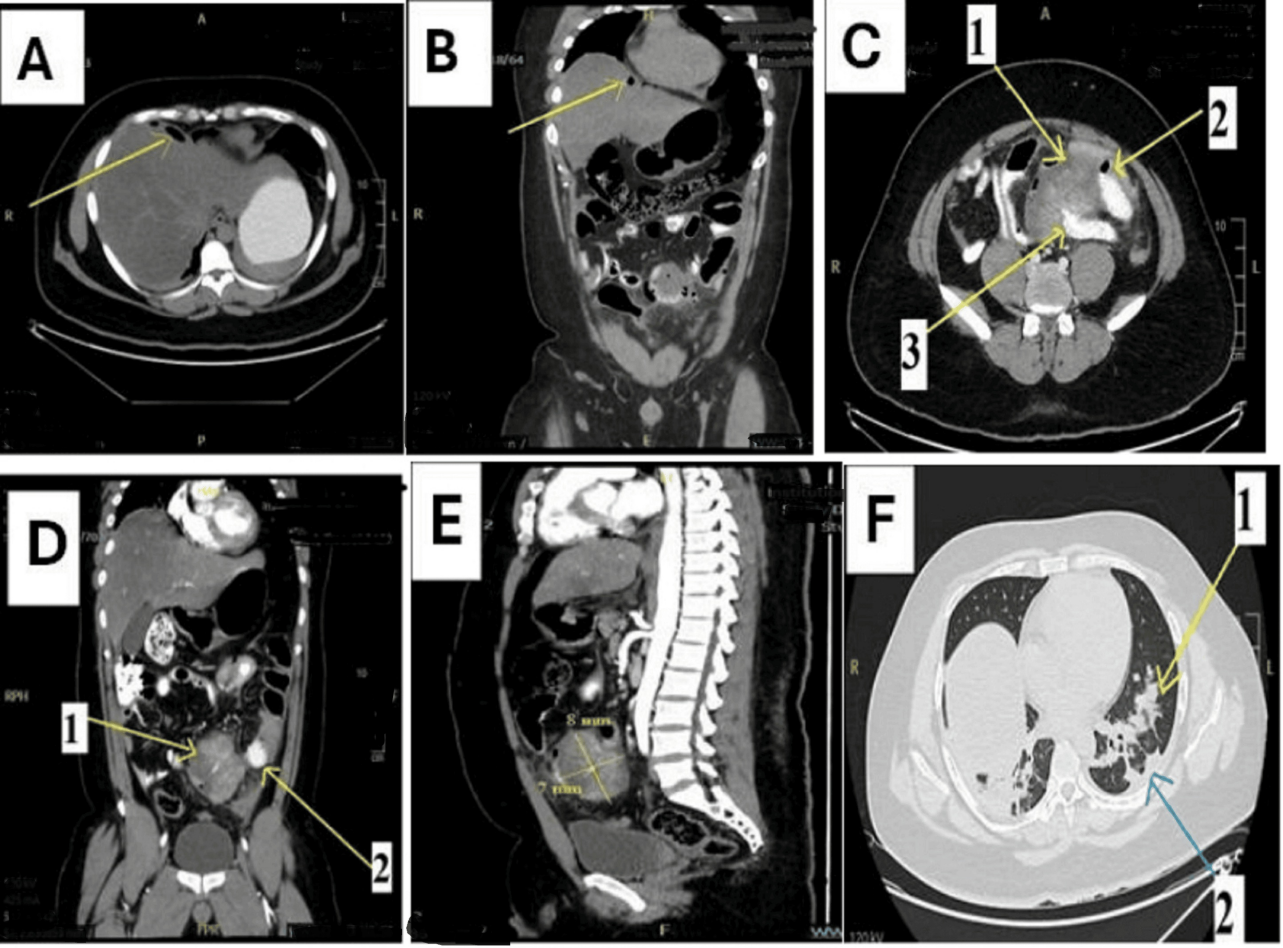
Contrast-enhanced CT of the abdomen (A, B) Axial and coronal views showing pneumoperitoneum (arrows). (C) Axial view indicating the tumor (arrow 1) and jejunum entry/exit sites (arrows 2, 3). (D) Coronal view identifying the GIST tumor (arrow 1) and jejunum (arrow 2). (E) Sagittal view illustrating tumor dimensions. (F) Axial view showing lower lobe consolidation (arrow 1) and pleural effusion (arrow 2). GIST: gastrointestinal stromal tumor

A contrast-enhanced computed tomography (CT) scan of the abdomen revealed pneumoperitoneum (Figures 1A-1B). A large, heterogeneously enhancing intra-peritoneal soft tissue mass, measuring 7.5 x 8.3 x 2.5 cm, was identified centered on small bowel loops at the jejunoileal junction (Figures 1C-1D). Its exophytic growth and enhancement pattern were highly suspicious for a GIST (Figure 1E). No abdominal lymphadenopathy or hepatic lesions were noted. Scanned lung cuts demonstrated bilateral lower lobe consolidation with mild pleural effusion (Figure 1F). The definitive diagnosis was a perforated small bowel tumor, likely a GIST, causing acute peritonitis.

**Figure 2:**
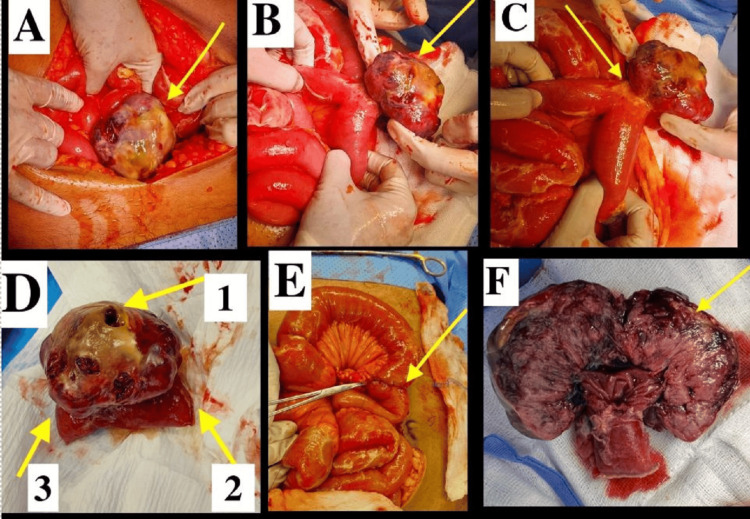
Intraoperative images (A) GIST tumor at laparotomy (arrow). (B) Tumor delivery into the operative field. (C) Tumor attached to the jejunal wall. (D) Arrow 1: perforation site; arrows 2 and 3: proximal and distal jejunum. (E) End-to-end anastomosis after resection (arrow). (F) Cut section of the specimen of the tumor. GIST: gastrointestinal stromal tumor

Following initial resuscitation with intravenous fluids and broad-spectrum antibiotics, the patient underwent an urgent exploratory laparotomy through a midline incision within eight hours of admission. Intraoperative exploration identified a perforated, lobulated 6 x 6 cm tumor originating from the antimesenteric border of the jejunum (Figures 2A-2D), located approximately 50 cm distal to the duodenojejunal junction. The peritoneal cavity contained minimal seropurulent fluid, and the remaining intra-abdominal viscera were inspected and found to be normal.

The involved segment of small bowel was resected with 5-cm margins proximal and distal to the tumor. An end-to-end anastomosis was performed using a two-layer hand-sewn technique with continuous 3-0 Vicryl sutures (Ethicon, Inc., Bridgewater, USA) (Figure 2E), and the mesenteric defect was closed with 3-0 Vicryl. Following confirmation of anastomotic integrity and saline irrigation of the peritoneal cavity, a size 16 drain was placed. The abdomen was closed using a mass closure technique with continuous No. 1 nylon suture, and a negative pressure wound system was applied postoperatively. The cut section of the specimen revealed a solid tumor (Figure 2F), which was sent for histopathological evaluation.

**Figure 3:**
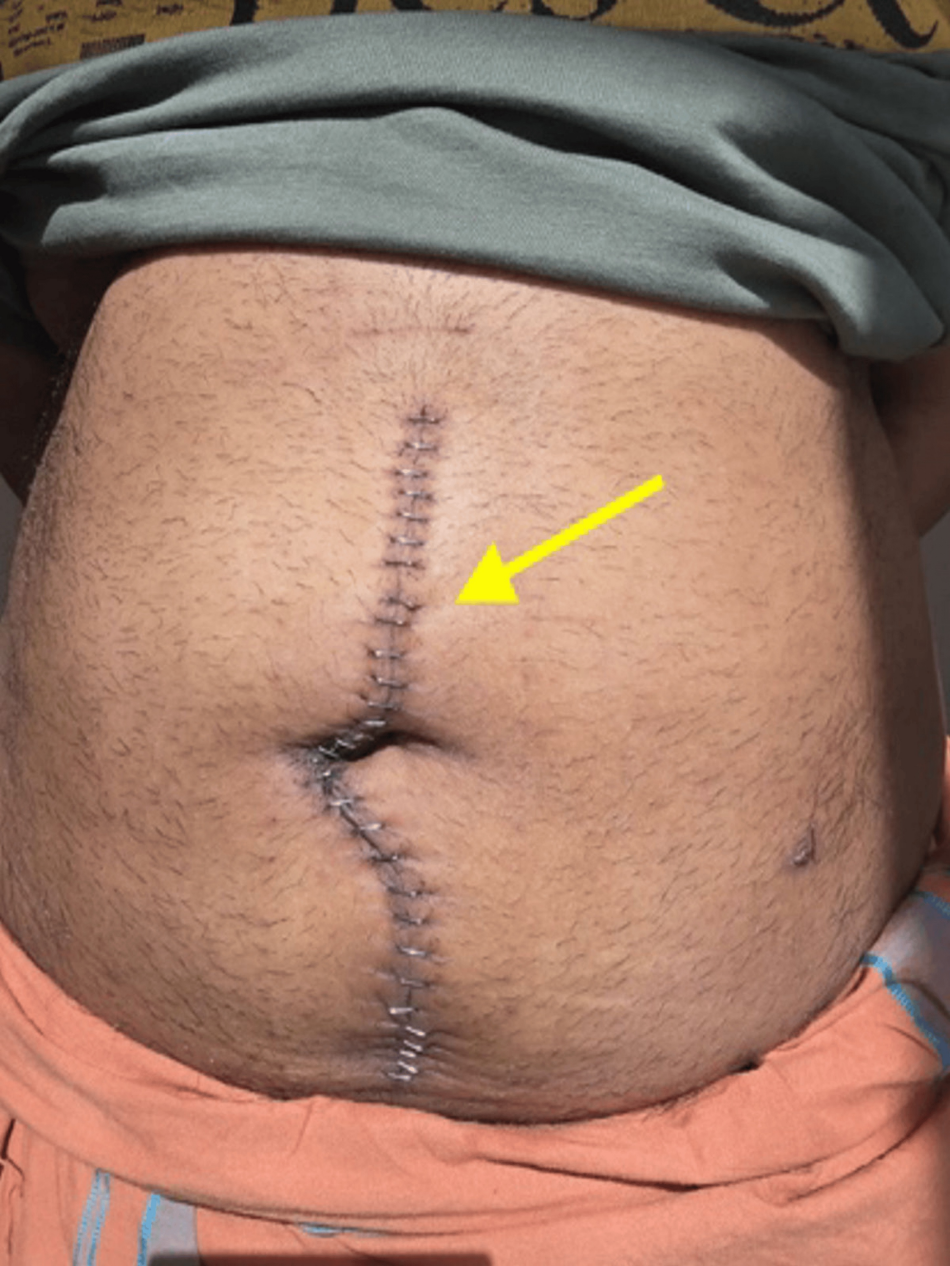
Postoperative image with an arrow indicating the well-healed laparotomy scar

The patient was managed postoperatively in the intensive care unit, requiring high-flow oxygen therapy for several days due to low oxygen saturation, with gradual improvement. Oral feeding was resumed gradually, the abdominal drain was removed on the third postoperative day, and the patient was discharged on postoperative day 6 in good condition. A follow-up examination at two weeks revealed a completely healed abdominal wound with no abdominal complaints (Figure 3). The patient was referred to an oncologist for further evaluation regarding adjuvant therapy. A whole-body positron emission tomography-CT (PET-CT) scan performed four weeks after discharge revealed no abnormal fluorodeoxyglucose (FDG) concentration, confirming the absence of residual disease or metastasis.

Histopathology confirmed the diagnosis of a high-grade GIST of the spindle cell type, which was unifocal with tumor necrosis and increased mitotic activity (>5/High Power Field). Oncogene analysis of the specimen by next-generation sequencing (NGS) revealed KIT EXON 11 deletion positivity and no mutation in PDGFRA genes. The patient was started on adjuvant imatinib therapy by the oncologist.

## Discussion

The term "GIST" was formally established in 1998 after the discovery of c-KIT (CD117) mutations by Hirota et al. [1]. Before this, many GISTs were misclassified as leiomyomas, leiomyosarcomas, or other spindle cell tumors. GISTs represent the most common mesenchymal tumors of the GI tract, comprising up to 80% of such neoplasms, while the remaining cases include leiomyomas, leiomyosarcomas, desmoid fibromatosis, and myofibroblastic tumors [1,2]. Although all GISTs have malignant potential, the risk of malignancy is primarily correlated with tumor size of 5 centimeters or more [2]. However, these tumors represent only 1-2% of all GI cancers. Arising from the muscularis propria, GISTs often remain undetected until they reach a significant size of 5 centimeters or more, potentially leading to complications such as chronic or acute bleeding, intestinal obstruction, or, in rare cases, perforation with subsequent peritonitis. Most cases are diagnosed in individuals aged 60 to 74 years, with the stomach (63%), small intestine (30%), rectum (3%), and esophagus (0.7%) being the most affected sites. Histopathological differentiation among cases shows that 38% are well-differentiated, 32% moderately differentiated, 19% undifferentiated, and 12% poorly differentiated [3]. Small, incidental GISTs, also known as tumorlets, are frequently identified during imaging, endoscopic evaluations, abdominal surgeries, or post-mortem examinations [3].

Jejunal GIST perforation is an uncommon occurrence, particularly among younger patients under 40 years old. In a large case series, only 6 out of 52 reported GIST cases of young individuals, which were presented as acute abdomen with small bowel location of the tumor, were presented in perforation, with KIT and PDGFRA mutation detected in 75% cases [4]. The literature classifies GIST rupture into three distinct types: closed perforation forming an abscess, rupture of a hematoma capsule leading to hemoperitoneum, and full-thickness bowel perforation [4-6]. Contrast-enhanced abdominal CT remains the gold standard for diagnosing this condition [7].

Timely detection necessitates urgent surgical intervention, typically involving resection of the affected jejunal segment, to reduce morbidity and mortality [8]. Both laparoscopic and open surgical approaches have been described for managing perforated cases. If laparoscopy is selected, adherence to oncologic principles is critical [8,9]. However, several studies advise against laparoscopic surgery for large, perforated tumors (≥5 cm) due to risks of inadequate tumor handling, rupture, and tumor cell spillage [8]. In such cases, exploratory laparotomy is generally preferred [10,11]. The standard treatment for localized GIST involves complete surgical removal with a macroscopic margin of at least 1 cm, while taking care to avoid tumor rupture during the procedure [11]. The number of mitotic figures (MF) per 5 mm^2^, in relation to the tumor's size in cm, is considered a histopathological predictor of recurrence. Tumors with fewer than 5 MF/mm^2^ and a size smaller than 2 cm are associated with the lowest risk of recurrence, whereas tumors with more than 5 MF/mm^2^ and a size between 2 and 5 cm carry a 70% risk. Tumors larger than 5 cm are linked to a recurrence rate exceeding 90% [11].

The risk of recurrence is significantly high for patients experiencing GIST rupture into the abdominal cavity, approaching nearly 100% [12]. Tumor rupture, whether spontaneous or intraoperative, is classified as metastatic disease and necessitates adjuvant imatinib therapy. The duration of imatinib treatment is individualized based on patient-specific factors [11,12]. Standard first-line therapy consists of 400 mg of imatinib daily, while patients with a KIT exon 9 mutation are advised to take 400 mg twice daily [11]. A recent 10-year follow-up from the SSGXVIII/AIO trial reinforced the long-term survival benefit of a three-year imatinib regimen, showing an overall survival rate of 79% compared to 65% in those receiving only 12 months of treatment [13].

Recurrence rates are highest within the first five years post-surgery (approximately 70%), increasing to 90% within 10 years and 95% within 15 years, with late recurrences beyond 20 years being rare [13,14]. Due to their high-risk classification, perforated GISTs require stringent follow-up. The National Comprehensive Cancer Network (NCCN) recommends a surveillance protocol with CT scans every four months during the first one to two years, every six months from years 3-5, and annually thereafter for high-risk patients [14,15].

Our literature review across PubMed and Google Scholar revealed around 36 case reports of jejunal GIST perforations, among which only five cases were 40 years or younger, and details of each case management are summarized in Table 1.

**Table 1:** Summary of reported cases of perforated jejunal GIST in patients aged ≤40 years, compiled from referenced sources (data not directly reproduced) N/A: information not available; GIST: gastrointestinal stromal tumor

S. No.	Author	Age in Years	Sex	Surgery Procedure	Adjuvant Imatinib	Follow-Up and Outcome
1	Manoharan et al. [16]	40	Male	Laparotomy with resection and primary side-to-side anastomosis	Yes	N/A
2	Ku et al. [17]	33	Female	Laparotomy with resection of the bowel	N/A	N/A
3	Hota et al. [18]	36	Male	Laparotomy with resection of the jejunal mass	N/A	Three months, no recurrence
4	Hans et al. [19]	40	Male	Resection	Yes	N/A
5	Choudhary et al. [20]	35	Male	Jejunum laparotomy with resection and end-to-end anastomosis	N/A	Two years, no recurrence

## Conclusions

Perforated jejunal GISTs leading to acute peritonitis are uncommon in individuals under 40 years of age. In our case, a prompt diagnosis was achieved using a contrast-enhanced CT scan of the abdomen, which warranted immediate surgical intervention. The affected bowel segment was resected with an adequate margin, resulting in a favorable clinical outcome. Laparoscopic surgery is not recommended for GIST tumors larger than 5 cm; accordingly, we opted for a laparotomy approach. Since spontaneous perforated GISTs are considered advanced disease, adjuvant chemotherapy with imatinib for up to three years is required. This should be accompanied by close follow-up using contrast-enhanced CT scans to monitor for recurrence and metastasis over the same period.
